# Expression of goat poxvirus P32 protein and monoclonal antibody preparation

**DOI:** 10.3389/fcimb.2024.1427588

**Published:** 2024-09-10

**Authors:** Ying Liu, Lei Wang, Xiaoyun Chen, Xin Ma, Chunsheng Yin, Chenghuai Yang, Bo Liu, Jige Du

**Affiliations:** ^1^ China Institute of Veterinary Drug Control, Beijing, China; ^2^ Joint FAO/IAEA Centre of Nuclear Techniques in Food and Agriculture, International Atomic Energy Agency, Vienna, Austria

**Keywords:** goat pox virus, P32 protein, soluble expression, monoclonal antibody, indirect immunofluorescence

## Abstract

P32 protein serves as a crucial structural component of Goat pox virus (GTPV), which causes a highly virulent infectious disease in sheep and goats. Despite the fact that P32 has been widely expressed in the previous studies, it is difficult to obtain recombinant P32 efficiently. This study aimed to achieve soluble expression of P32 recombinant protein and to develop its specific monoclonal antibody. The gene fragment of P32Δ (GP32Δ) was synthesized by optimizing the coding sequence of amino acids 1-246 of the known goatpox P32 protein. Subsequently, GP32Δ was cloned into a prokaryotic expression vector for expression and purification, resulting in the successful production of soluble recombinant protein rP32Δ. Utilizing rP32Δ, an indirect ELISA method was established by immunizing 6-week-old BALB/c mice with inactivated GTPV as the antigen. Through hybridoma technology, three monoclonal antibody hybridoma cell lines secreting anti-goat pox virus rP32Δ were screened, designated as 2F3, 3E8, and 4H5, respectively. These monoclonal antibodies, classified as IgG1, IgG2a, and IgG2b, respectively, with κappa light chains, were characterized following ascites preparation and purification. Indirect ELISA results demonstrated that the ELISA potency of the three monoclonal antibodies exceeded 1:12800. Furthermore, Western blot analysis revealed specific reactivity of both 3E8 and 4H5 with rP32Δ, while immunofluorescence assays confirmed 3E8's ability to specifically recognize GTPV in cells. The preceding findings demonstrate the successful acquisition of the soluble expressed recombinant P32 protein and its specific monoclonal antibody 3E8 in this study, thereby laying a foundational material basis for the establishment of a GTPV detection method.

## Introduction

Goat pox virus (GTPV) is a highly virulent infectious disease causing malignant pox (goat pox) in sheep and goats, categorized as a class A disease by the World Organization for Animal Health (WOAH). Infection with GTPV manifests in clinical symptoms such as fever, lethargy, weight loss, and decreased milk production in affected animals (Babiuk et al., 2008). It exhibits a significant morbidity rate (75-100% in endemic areas) (Bhanuprakash et al., 2006) and mortality rate (10-85%), particularly among susceptible animals like lambs, with mortality rates reaching close to 100%, resulting in substantial economic losses (Zewdie et al., 2021). GTPV infections are widespread, and prevalent in Central Africa, the Middle East, Europe, and Asia, posing serious threats to the healthy development of the sheep farming industry (Hamdi et al., 2021a; Garner et al., 2000; Suresh et al., 2022; Yan et al., 2012). The disease also occurs in Gansu, Ningxia, and Hubei provinces within China (Zhou et al., 2012). With the introduction and movement of breeds in goat farming, the spread and prevalence of goat pox are concerning (Tadesse et al., 2022). Hence, there is an urgent need to enhance the detection, prevention, and control measures against GTPV.

Currently, the prevention and control of GTPV primarily rely on goat pox vaccines (Hamdi et al., 2021b). Despite a few reports on the GTPV antigen/antibody detection (Babiuk et al., 2009; Bowden et al., 2009; Ebrahimi-Jam et al., 2021; Heine et al., 1999; Lamien et al., 2011), the evaluation of immune responses in vaccinated animals is predominantly conducted using the Virus Neutralization Test (VNT), which involves live GTPV and poses significant biosafety risks (Yan and Zhang, 2009). Consequently, there is an urgent demand for the establishment of convenient and effective GTPV antigen/antibody detection methods. Previously, both recombinant virus proteins-based ELISA and inactivated virus-based ELISA was used to detect the capripoxviruses-specific antibodies (Babiuk et al., 2009; Bowden et al., 2009; Heine et al., 1999; Venkatesan et al., 2018). However, it is expensive and difficult to produce the large quantities of viral antigen needed for serological surveillance (Babiuk et al., 2009).Therefore, the recombinant virus proteins-based ELISA was an ideal assay. Among these recombinant virus proteins (ORF 095, ORF 103 and ORF 070, also known as P32), P32 protein serves as a crucial structural component of GTPV and used widely to developed indirect ELISA system (Tulman et al., 2002).However, the exogenous expression of P32 poses challenges (Song et al., 2019; Zhang et al., 2017). Hence, efficient expression and purification of recombinant P32 protein are pivotal for elucidating GTPV pathogenesis and establishing detection methods. In this study, we aimed to optimize the codon sequence of the selected GTPV truncated P32 protein (amino acids 1-246), followed by prokaryotic expression, and screening and application studies of monoclonal antibodies against recombinant P32 protein in conjunction with GTPV.

## Materials and methods

### Cells, virus strains, plasmids, experimental animals, and reagents

The SP2/0 myeloma cell line and sheep pox virus (AV41) were maintained in our laboratory. SPF-grade female 6-week-old BALB/c mice and reproductively experienced female BALB/c mice were procured from Beijing Vital River Laboratory Animal Technology. All mice were euthanized by cervical dislocation at the end of the experiment. Furthermore, procedures for immunization and use of mice in antibody production were approved by the Animal Care and Use Committee of China Institute of Veterinary Drug Control. Protein Marker and Western blot Marker were sourced from Gen Script (Nanjing). The pEASY-Blunt Cloning Vector, receptor cells Top10 and BL21 (DE3) were obtained from TransGen Biotech (Beijing). MEM culture medium was purchased from Gibco, while fetal bovine serum was sourced from PAN. Polyethylene Glycol (PEG1450), Fuchs’ Adjuvant, HAT, and HT Selective Medium were acquired from Sigma. Mab isotype identification kits were obtained from Biodragon (Beijing). High-fidelity PCR enzyme (KFX-401S) was procured from Toyobo, while premix Taq version 2.0 and DNA Marker were purchased from Takara. T4 ligase and DNA gel recovery kit were obtained from Promega, and restriction endonucleases *Kpn*I and *Xho*I were sourced from NEB. The anti-His monoclonal antibody and Bradford protein concentration kit were sourced from Beyotime Biotechnology. HRP-labeled goat anti-mouse IgG was obtained from ZSGB-BIO.

### Construction of prokaryotic expression vectors

For the construction of prokaryotic expression vectors, the amino acid coding sequence (amino acid positions 1-246) of the GTPV P32 protein (Genbank: MG458384.1) was optimized to match the preferred codon usage of *Escherichia coli*. Additionally, a 6× His tag was incorporated at the C-terminal end of the sequence. Subsequently, the gene fragment P32Δ (GP32Δ) was synthesized chemically, and then inserted into the pET-30a (+) vector to generate the pET-GP32Δ construct.

### Expression and purification of recombinant proteins

The expression and purification of recombinant proteins followed the protocol outlined by Chen et al (Chen et al., 2018). Briefly, BL21(DE3) receptor cells were separately transformed with the pET-GP32Δ and pET-30a (+) constructs. Expression was induced using IPTG at 15°C and 37°C for 16 hours and 4 hours, respectively. Following induction, bacterial cultures were harvested and subjected to ultrasonication to collect both the supernatant and precipitate fractions. The expression and solubility of recombinant proteins were assessed via SDS-PAGE. Subsequently, rP32Δ was purified from the cell supernatants according to the method described by Chen et al (Chen et al., 2018). The protein concentration was determined using a Bradford protein concentration kit, and the purified proteins were stored at -80°C.

### Animal immunization

Five female BALB/c mice were immunized with inactivated sheep pox virus as the immunogen via subcutaneous multipoint injection, with a dose of 10^4.9^ TCID_50_ per mouse. Immunizations were administered every 14 days. 7-10 days after the third immunization, blood samples were collected from the orbital region, and the serum potency was assessed via indirect ELISA. If the serum potency exceeded the required threshold for fusion (potency higher than 12 800), the mice received reinforcement three days prior to fusion through direct intraperitoneal injection of rP32Δ protein at a dose of 50 μg per animal. The immunization protocol is outlined in **Table 1**.

**Table 1:** Immunization procedures for mice.

Number of Immunizations	Immunization route	Immunization dose (TCID_50_ per mouse or μg per mouse)
The first immunization	Multipoint subcutaneous injections in the back and neck	10^4.9^ TCID_50_
The second immunization	Multipoint subcutaneous injections in the back and neck	10^4.9^ TCID_50_
The third immunization	Multipoint subcutaneous injections in the back and neck	10^4.9^ TCID_50_
Strengthen immunization	Intraperitoneal injection (recombinant protein)	50 μg

### Cell fusion and detection

Splenocytes obtained from immunized mice were fused with SP2/0 cells at a ratio of 10:1 in the presence of PEG1450. The following specific method was employed: First, a 50 mL centrifuge tube was used to mix and centrifuge the cells, followed by discarding the supernatant. Subsequently, 1 ml of preheated PEG1450 at 37°C was added, and the centrifuge tube was gently spun. Then, 10 ml of serum-free DMEM medium was slowly added, followed by centrifugation and discarding of the supernatant. The cells were resuspended with HAT medium containing 20% fetal bovine serum and then spread onto 96-well cell culture plates containing feeder cells. Finally, the cell culture plates were placed into an incubator for cultivation. Hybridoma cells showing positive reactivity with rP32Δ were selected through indirect ELISA, followed by subcloning, construction, and expansion using the limited dilution method. Subsequently, the selected hybridoma cells were stored in liquid nitrogen for long-term preservation.

### Identification of antibody isotypes secreted by hybridoma cells

The isotypes of monoclonal antibodies were identified using hybridoma cell culture supernatants, following the procedure outlined in the instruction manual provided with the monoclonal antibody isotype identification kit from Biodragon (Beijing).

### Monoclonal antibody preparation

Preparation and purification of monoclonal antibody ascites and determination of indirect ELISA potency. Healthy reproductively experienced female BALB/c mice were chosen and administered intraperitoneally with 0.5 ml of Fuchs’ incomplete adjuvant. Ten days later, hybridoma cells were intraperitoneally injected into the mice, with approximately 10^6.0^ cells injected per mouse. Ascites were aseptically extracted when the abdominal circumference of the mice significantly enlarged, and they became immobile. Protein A (GE Healthcare 17-5079-01) affinity column purification method was employed for purification. The potency of monoclonal antibodies was assessed using indirect ELISA, encapsulating rP32Δ, the His-tagged control protein, and recombinant *Clostridium septicum* alpha toxin (CSA), rCSA (referred to as rCSA-His), following the methodology described by Chen et al (Chen et al., 2018).

### Western blot identification of monoclonal antibody reactivity

rP32Δ and rCSA-His underwent SDS-PAGE electrophoresis and were transferred onto a PVDF membrane (50V, 2 h), followed by blocking with 5% skimmed milk for 90 min. The membrane was then incubated with the purified monoclonal antibody (diluted 1:1 500) for 1.0 h at 37°, washed with PBST, and subsequently incubated with HRP-goat anti-mouse IgG (diluted 1:5 000) for 0.5 h at room temperature. After washing, the protein bands were detected using chemiluminescence detection.

### Immunofluorescence for identifying monoclonal antibody reactivity

Primary goat testis cells were infected with GTPV. After lesions developed in the virus-receiving cells, they were fixed with pre-cooled 80% acetone solution at room temperature for 15 minutes, followed by treatment with 0.5% TritonX-100 for 10 minutes. Subsequently, the cells were blocked with PBS containing 5% BSA at 37°C for 1 hour. Next, they were incubated with a monoclonal antibody (diluted 1:200) as the primary antibody at 37°C for 1 hour. After washing three times with PBST, the cells were incubated with FITC-labelled anti-mouse IgG (diluted 1:500) for 1 hour at 37°C. Following another three washes with PBST, the cells were observed under an inverted fluorescence microscope.

## Results

### Expression and characterization of rP32Δ

**Figure 1:**
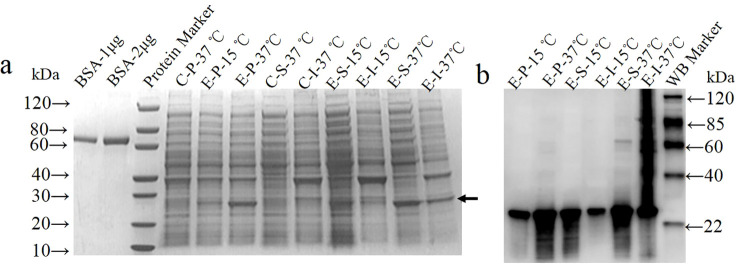
Expression and identification of rP32Δ. BL21(DE3)/pET30a (control group, C) and BL21(DE3)/pET-GP32Δ (experimental group, E) were induced with 0.5 mM IPTG at 37°C and 15°C, separately. The cell pellet (P), soluble (S) and insoluble fractions (I) were harvested and subjected to SDS-PAGE **(A)** and Western blot probed with anti-His (1:1000) **(B)**. The arrow shows rP32Δ.

The coding sequence of amino acids 1-246 of the GTPV P32 protein was synthesized and cloned into a prokaryotic expression vector. The recombinant expression plasmids pET30a-GP32Δ (referred to as pGP32Δ) and the empty vector pET-30a (+) (referred to as pET) were separately transformed into BL21(DE3) competent cells and induced for expression. SDS-PAGE (**Figure 1A**) and Western blot (**Figure 1B**) analysis revealed that the recombinant protein rP32Δ was expressed under both 15°C and 37°C induction conditions, with a molecular weight of approximately 29.5 kDa, consistent with the expected size. Expression of rP32Δ was lower at 15°C, predominantly in the form of inclusion bodies, while expression levels and solubility were higher at 37°C. Therefore, the supernatant of lysate induced at 37°C was selected for purification.

### Identification of rP32Δ

**Figure 2:**
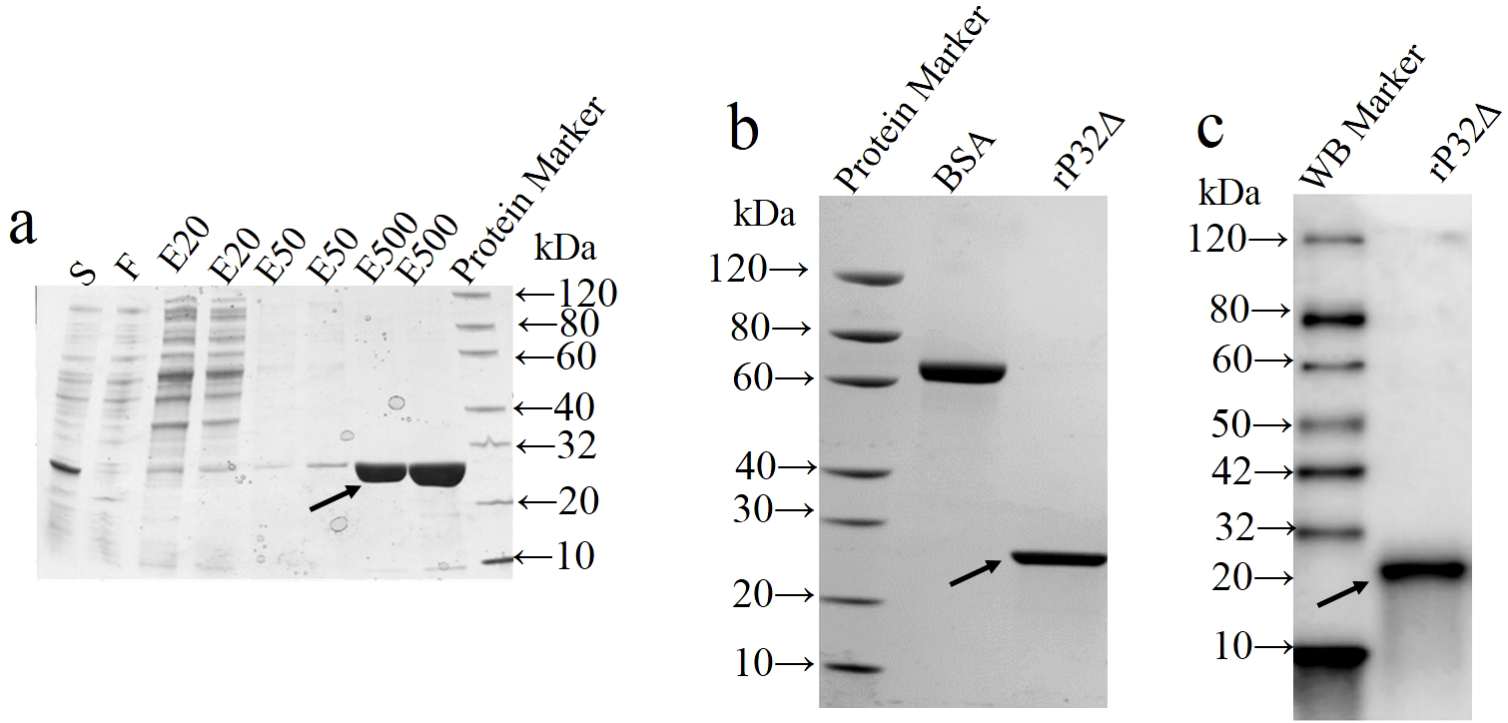
Purification and identification of recombinant protein. The supernatant (S) of cell lysates was loaded onto the Ni resin and the flow-through (F) from Ni-IDA resin was collected. Unbound protein was removed by washing. rP32Δ was eluted with elution buffer containing different concentrations of imidazole at 20 (EB-20), 50 (EB-50) and 500 (EB-500) mM, and then samples were subjected to SDS-PAGE **(A)**. After dialysis and concentration, purified proteins were validated by SDS-PAGE **(B)** and Western blot **(C)**. Arrows show rP32Δ.

As shown in **Figure 2A**, rP32Δ was purified using Ni-IDA affinity chromatography according to the manufacturer’s instructions. The eluate with high purity (elution buffer containing 500 mM imidazole) was collected and subjected to dialysis. The final protein concentration obtained was 1.02 mg/ml, with a purity of over 92% (**Figure 2B**). Additionally, it exhibited specific reactivity with anti-His tagged protein monoclonal antibodies (**Figure 2C**).

### Screening of hybridoma cells and identification of monoclonal antibody isotypes.

Following cell fusion, positive hybridoma cell clones reacting with rP32Δ were screened via indirect ELISA. After three rounds of subcloning, three hybridoma cell lines were established, capable of stable secretion of monoclonal antibodies against rP32Δ, designated as 2F3, 3E8, and 4H5, respectively. Antibody subtyping (**Table 2**) revealed that the monoclonal antibodies produced by these cell lines belonged to the IgG1, IgG2a, and IgG2b isotypes, with all light chains being of the κ type.

**Table 2:** Identification of monoclonal antibody isotypes.

Monoclonal antibody	Light chain	Isotype
2F3	κ	IgG1
3E8	κ	IgG2b
4H5	κ	IgG2a

### Purification of monoclonal antibodies and determination of indirect ELISA potency

**Figure 3:**
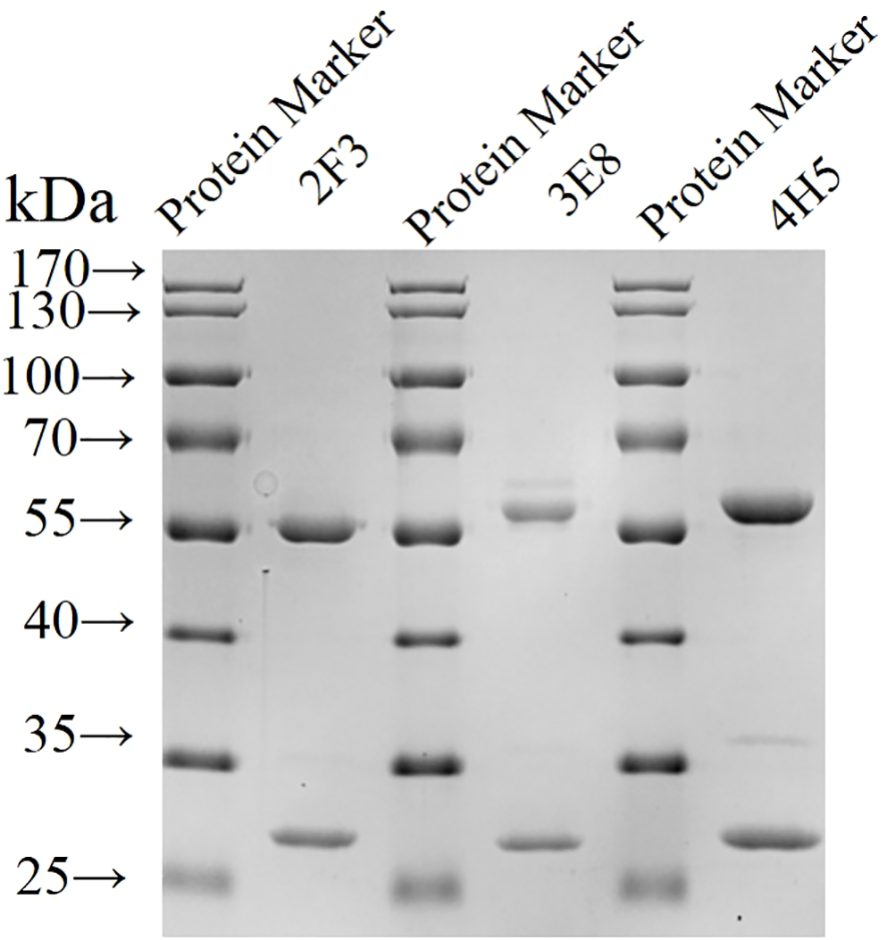
Identification of purified monoclonal antibodies by SDS-PAGE Monoclonal antibodies were purified and subjected to SDS-PAGE.

**Figure 4:**
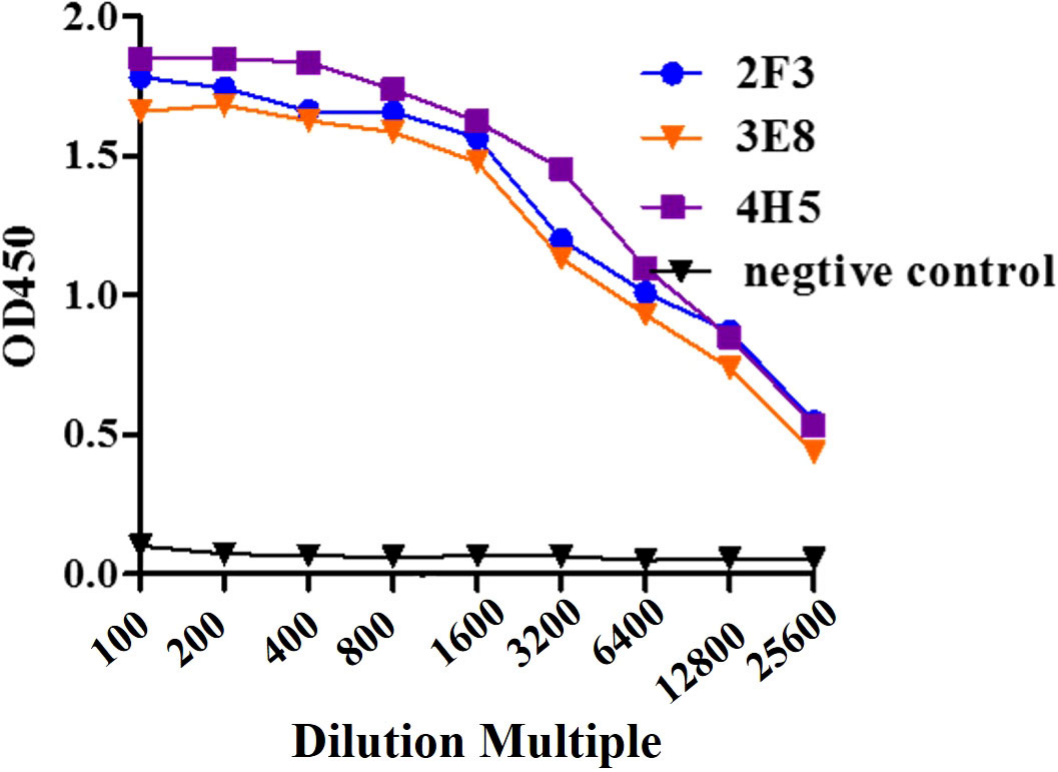
Sensitivity assay. Monoclonal antibody 2F3, 3E8 and 4H5 with 2-fold dilutions from 1:100 to 1:51 200 were detected with ELISA.

Three hybridoma cell lines mentioned above were expanded, and monoclonal antibodies 2F3 (mAb-2F3), 3E8 (mAb-3E8), and 4H5 (mAb-4H5) were purified following the preparation of mouse ascites. The purity of the three monoclonal antibodies exceeded 90% (**Figure 3**), with concentrations of 2.1 mg/mL, 2.0 mg/mL, and 3.8 mg/mL, respectively. Indirect ELISA results demonstrated that none of the three monoclonal antibodies exhibited reactivity with rCSA-His, while their potency in indirect ELISA reactions with rP32Δ exceeded 1:12 800 (**Figure 4**).

### Western blot identification of monoclonal antibodies reacting with rP32Δ

**Figure 5:**
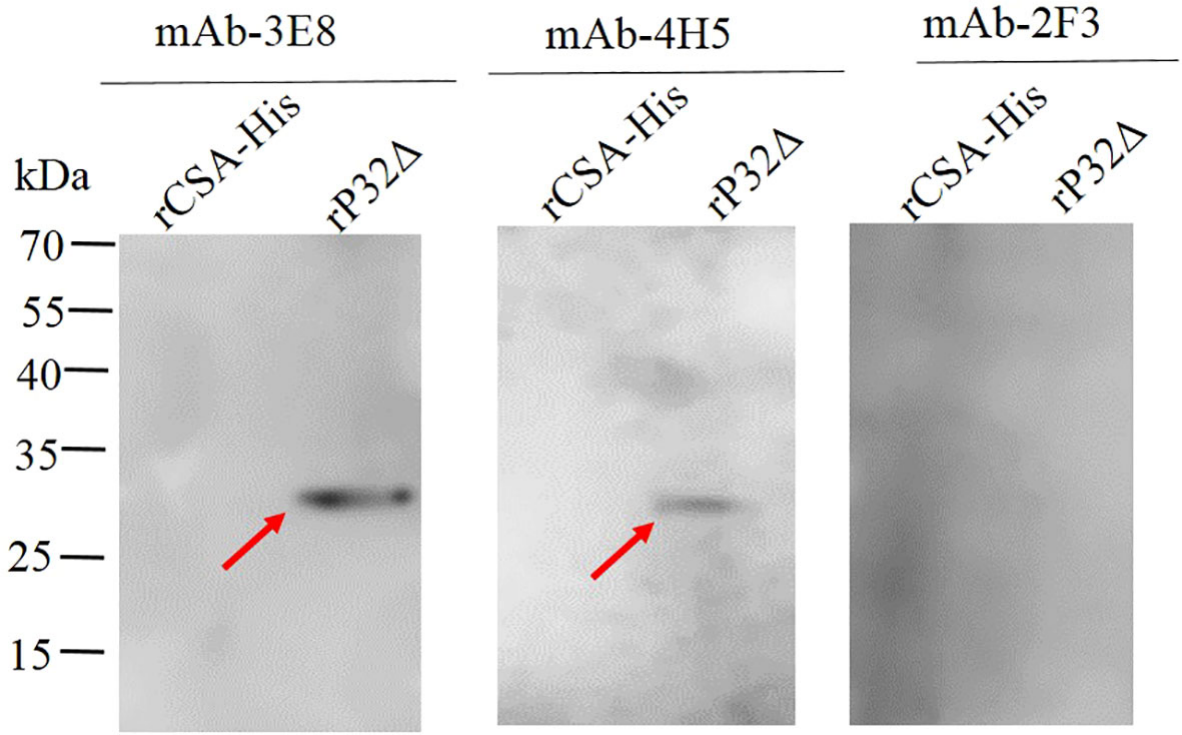
The identification of purified monoclonal antibodies by Western blot. The rP32Δ and His-tagged control protein (rCSA-His) were subjected to SDS-PAGE and Western blot probed with monoclonal antibodies 2F3, 3E8 and 4H5, respectively. Arrows show rP32Δ.

To further confirm the specificity of the monoclonal antibodies, Western blot analysis was conducted to assess their reactivity with rP32Δ and a protein control with a His tag (rCSA-His). The results of the Western blot demonstrated that mAb-3E8 and mAb-4H5 exhibited specific reactivity with rP32Δ, generating distinct bands at 29.5 kDa. In contrast, mAb-2F3 did not display a specific band when reacting with rP32Δ. Moreover, none of the three monoclonal antibodies showed specific reactivity with rCSA-His (**Figure 5**).

### Monoclonal antibody 3E8 specifically reacts with GTPV by immunofluorescence

**Figure 6:**
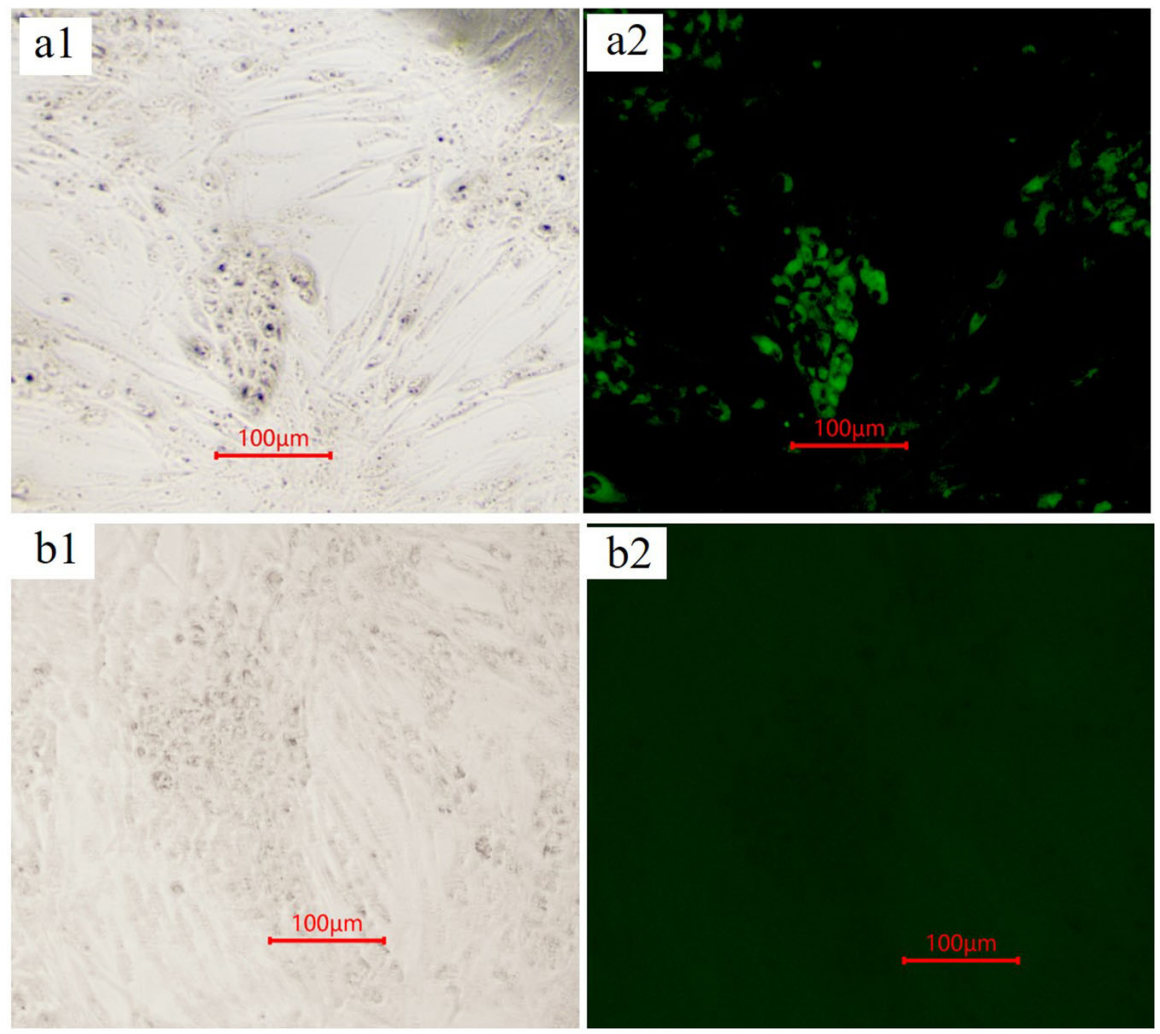
The specific interaction monoclonal antibody 3E8 and GTPV identified by indirect immunofluorescence. The Primary Goat Testicular cells infected by GTPV **(A1)** and Cell control **(B1)** were fixed, permeabilized, stained with monoclonal antibody 3E8, and examined by fluorescence microscope (**A2** and **B2**).

The reactivity of the aforementioned monoclonal antibodies with GTPV was evaluated using indirect immunofluorescence (IFA). The results of the IFA demonstrated that mAb-3E8 specifically recognized GTPV in primary goat testis cells (**Figure 6**). Conversely, neither mAb-2F3 nor mAb-4H5 exhibited specific recognition of GTPV in goat testis cells (results not shown).

## Discussion

Although the virus neutralization test (VNT) with high specificity is considered the gold standard for laboratory diagnosis of capripoxvirus, it poses significant biosafety risks and may not detect low levels of antibodies from vaccinated animals (Zewdie et al., 2021). Recombinant viral proteins and monoclonal antibodies based-ELISA is one of the most feasible assays being used in antigen/antibody of virus. Therefore, this study was conducted to obtain the crucial recombinant protein of GTPV and monoclonal antibodies, which may be used to develop an ELISA assay in antigen/antibody of GTPV.

Zhang et al. reported successful expression of a fusion protein (GST-P32) consisting of glutathione-S-transferase (GST) and the P32 protein (lacking the transmembrane region) using a prokaryotic system in 2007; however, GST-P32 expression levels were low and purification was not achieved (Zhang et al., 2007). Similarly, Song et al. attempted expression of the full-length P32 gene, the gene encoding the extramembranous region of P32 (P321-282), and the gene encoding the extramembranous hydrophilic region of P32 (P3220-270) in prokaryotic expression plasmids (pET42b). They found that while full-length P32 expression was challenging in *E. coli*, the extramembrane region could be expressed albeit at low levels and with difficulty in purification (Song et al., 2019). This may be attributed to the toxic transmembrane helical structures present in amino acids 287 to 307 at the C-terminal end of the complete P32 protein, as well as the presence of rare codons in the P32 gene unfavorable for expression in *E. coli* (Zhang et al., 2007). Recent findings by Ebrahimi-Jam et al. support these results, as they attempted expression of full-length P32 protein (1-322 aa) and truncated P32 protein (20-270 aa) in *E. coli* expression bacteria (Rosetta), with no detection of full-length P32 protein and predominant expression of truncated P32 protein as inclusion bodies (Ebrahimi-Jam et al., 2021). Although full-length P32 protein has been successfully expressed in BHK-21 cells and the Pichia pastoris system has been used for P32 protein expression, the eukaryotic expression system is economically more costly than the prokaryotic expression system (Bhanot et al., 2009; Chen et al., 2008). In this study, the region spanning amino acid positions 1 to 246 of the P32 protein was selected for expression, and the gene fragment was optimally designed and artificially synthesized based on *E. coli*-preferred codons. A truncated recombinant protein of the P32 protein, rP32Δ, was successfully expressed in the prokaryotic system with a high ratio of soluble expression, and ultimately purified in a nondenaturing form with a high concentration.

Monoclonal antibody preparation is essential for establishing reliable antigen/antibody detection methods, with the selection of immunogens being a key factor. A monoclonal antibody specific for capripoxvirus, which was generated to *Escherichia coli*-expressed sheeppoxvirus ORF 057, have been used in immunohistochemistry to characterize the tissue and cell tropism of sheeppox and goatpox viruses (Embury-Hyatt et al., 2012). Considering that P32 protein was a crucial structural component of GTPV and used as diagnostic antigen in indirect ELISA (Tulman et al., 2002), it is necessary to obtain anti-P32 monoclonal antibodies, which can be used widely in laboratory diagnosis. A GTPV-P32 protein specific monoclonal antibody was used in Western blot to detect the expression of recombinant P32 in *Pichia pastoris*, however, it was not evaluated in other antigen/antibody detection methods (Bhanot et al., 2009). Therefore, our present study was conducted to get the sensitive, specific and widely used monoclonal antibodies against P32. Given the challenges in expressing full-length P32 protein in prokaryotic systems, a truncated recombinant P32 protein was obtained in this study, lacking the post-translational modifications of eukaryotic expression systems. Using rP32Δ as both the immunogen and encapsidated antigen in indirect ELISA greatly facilitated the generation of monoclonal antibodies specifically targeting rP32Δ without cross-reactivity with the natural GTPV virus. To achieve this, mice were pre-immunized with inactivated goat pox vaccine for robust immunoprotection, followed by booster immunization with rP32Δ protein stimulation prior to hybridoma cell fusion. The use of rP32Δ as the encapsidated antigen for positive cell screening enabled the establishment of an indirect ELISA method. Additionally, employing both GTPV and rP32Δ for anti-GTPV monoclonal antibody screening ensured maximum specificity and ease of reproducibility. In this study, recombinant *Clostridium septicum* alpha toxin with a His tag served as a control in indirect ELISA experiments, excluding interference from the His tag and confirming the reactivity of all three monoclonal antibodies with rP32Δ. Subsequent Western blot and immunofluorescence assays demonstrated that mAb-3E8 exhibited broader reactivity. Further research investment is required in the later stages to analyze and obtain information on the antigenic epitopes targeted by mAb-3E8 for optimal utilization in immunohistochemistry and antigen/antibody serological assay for GTPV.

## Data Availability

The original contributions presented in the study are included in the article/supplementary material. Further inquiries can be directed to the corresponding authors.
